# PTH Analog Therapy in CKD G4–G5D: Current Evidence and Potential Role of Abaloparatide in Adynamic Bone Disease

**DOI:** 10.3390/jcm15010133

**Published:** 2025-12-24

**Authors:** Laia Gifre, Maria Fusaro, Maria J. Lloret, Elisabet Massó, Pilar Peris, Xavier Nogués, Rosana Gelpi, Águeda Prior-Español, Jordi Ara, Mario Cozzolino, Pablo A. Ureña-Torres, Jordi Bover

**Affiliations:** 1 Rheumatology Department, Hospital Germans Trias i Pujol, Research Institute Germans Trias i Pujol (IGTP), Universitat Autònoma de Barcelona, C/Canyet S/N, 08196 Badalona, Spain; 2 Department of Medicine, University of Padova, 35122 Padova, Italy; 3 Institute of Clinical Physiology (IFC), National Research Council (CNR), 00185 Pisa, Italy; 4 Nephrology Department, Fundació Puigvert, Institut de Recerca Sant Pau (IR Sant Pau), Universitat Autònoma de Barcelona, 08025 Barcelona, Spain; 5 REMAR-IGTP Group, RICORS 2040, Nephrology Department, Research Institute Germans Trias i Pujol (IGTP), University Hospital Germans Trias i Pujol, Universitat Autònoma de Barcelona, 08196 Badalona, Spainjbover.ics@gencat.cat (J.B.); 6 Rheumatology Department, Hospital Clínic Barcelona, Fundació de Recerca Clínic Barcelona-Institut d’Investigacions Biomèdiques August Pi i Sunyer (FRCB-IDIBAPS), Universitat de Barcelona, 08036 Barcelona, Spain; 7 Internal Medicine Department, Hospital del Mar Research Institute, CIBER de Envejecimiento y Fragilidad Saludable, Universitat Pompeu Fabra, 08003 Barcelona, Spain; 8 Renal Division, Department of Health Sciences, University of Milan, 20122 Milan, Italy; 9 Department of Nephrology and Dialysis, AURA Nord Saint-Ouen, 93400 Saint-Ouen, France; 10 Department of Renal Physiology, Necker Hospital, University of Paris Descartes, 75015 Paris, France

**Keywords:** chronic kidney disease, CKD-MBD, adynamic bone disease, osteoporosis, fragility fractures, bone turnover, bone-forming therapies, Abaloparatide

## Abstract

Osteoporosis and fragility fractures are among the most prevalent and clinically significant complications in patients with chronic kidney disease (CKD), particularly in stages G4–G5 and in those undergoing dialysis (G5D). These skeletal disorders are associated with markedly increased morbidity and mortality, including a 2- to 9-fold higher risk of hip fractures compared to the general population, prolonged hospitalization, functional decline, and excess postoperative mortality. Despite this substantial burden, CKD-associated osteoporosis remains underrecognized and undertreated. Limited inclusion of CKD patients in pivotal osteoporosis trials and the absence of high-evidence guidance in clinical guidelines have contributed to a persistent therapeutic gap. PTH analog agents such as teriparatide and abaloparatide have demonstrated robust efficacy in increasing bone mass and reducing fracture risk in the general population. However, their use in CKD remains limited. PTH analog are poorly prescribed in patients with CKD stage G3 and remain off-label for stages G4–G5D, despite the high prevalence of adynamic bone disease across all stages of CKD. Abaloparatide, a selective PTH1 receptor agonist, exerts potent anabolic effects with a lower incidence of hypercalcemia than teriparatide and may offer a favourable safety profile in carefully selected patients. Preliminary data suggest preservation of bone microarchitecture and potential benefits in low-turnover bone disease, although evidence in CKD is still limited. This narrative review examines current evidence on abaloparatide’s potential role in CKD, emphasizing its mechanism of action, efficacy, safety, and relevance for patients with low bone turnover and high fracture risk.

## 1. Introduction

It is well known that chronic kidney disease (CKD) profoundly disrupts mineral metabolism, giving rise to a spectrum of disorders collectively referred to as CKD-mineral and bone disorder (CKD-MBD) [1]. CKD-MBD encompasses abnormalities in mineral metabolism, bone structure, cardiovascular calcification, and it is strongly associated with increased morbidity, mainly from cardiovascular disease, and mortality [1]. Alterations in calcium, phosphate, parathyroid hormone (PTH), vitamin D and fibroblast growth factor-23 (FGF23), among others, emerging at different stages of CKD [2,3], lead to significant skeletal complications. The current staging of CKD is summarized in Table 1.

It has only recently been recognized that osteoporosis and fragility fractures are also common complications in patients with CKD. Up to 20–30% of CKD patients have densitometric osteoporosis, with prevalence rising to 50% among those on hemodialysis (G5D) [5,6]. However, osteoporosis in this population remains clearly underdiagnosed. Osteoporotic fractures are the primary consequence of bone fragility, and CKD patients have a well-documented 2- to 9-fold increased risk of fragility fractures compared to the general population—an association linked to increased morbidity and mortality [7,8].

Despite this, the 2009 KDIGO (Kidney Disease Improving Global Outcomes) guidelines did not recommend routine assessment of bone mineral density (BMD) in patients with CKD stages G3–G5D, based on moderate-quality evidence (2B) [9]. On the contrary, the updated 2017 KDIGO guidelines did suggest evaluating fracture risk (including BMD measurement) in these patients if the results would impact treatment decisions (also evidence 2B) [10,11]. More recently, the KDIGO 2025 controversies conference highlighted the urgent need to address the distinct yet overlapping entity of CKD-associated osteoporosis, calling for improved risk assessment strategies, greater inclusion of CKD patients in clinical trials, and updated therapeutic recommendations tailored to this population [12].

Although osteoporosis and fractures are prevalent in CKD patients, a significant treatment gap exists in their management. This therapeutic nihilism is partly due to a lack of robust scientific evidence, as patients with CKD stages G3–G5D have been largely excluded from most clinical trials evaluating antiosteoporotic agents [13]. The evidence gap is even more pronounced in dialysis populations. Moreover, as most available references are case series or post hoc analyses, it should be noted that the overall level of evidence in this field is very low (GRADE).

In fact, therapy for CKD-MBD has classically focused only on phosphate binders, different forms of vitamin D or calcimimetics, depending on the biochemical abnormalities (i.e., hyperphosphatemia, secondary hyperparathyroidism…) and/or the clinical suspicion of the underlying type of renal osteodystrophy (ROD). In this sense, different patterns of ROD are usually described: high-turnover osteitis fibrosa or mild hyperparathyroidism, low-turnover adynamic bone disease (ABD) and osteomalacia, and the mixed form named uremic osteodystrophy [1]. Bone-forming agents would be particularly useful in patients with low bone turnover, without bone mineralization defect, and would be further justified given that this condition is becoming increasingly common in dialysis patients.

In this context, there has been an increase in publications and case series exploring the utility of bone-forming agents, particularly teriparatide (a PTH 1–34 analog), in patients with CKD [14]. However, clinical evidence regarding the efficacy of the newly commercialized agents romosozumab and abaloparatide in this population remains scarce. Romosozumab is a humanized monoclonal antibody targeting sclerostin, which inhibits Wnt signaling [15]—a key pathway that regulates cell growth, differentiation, and tissue maintenance. Dysregulation of Wnt signaling has been linked to osteoporosis, cancer, and other diseases [16]. Abaloparatide is a synthetic analog of human parathyroid hormone-related protein that binds to the same receptor as teriparatide [17]. Since most studies on bone-forming agents in CKD have focused on teriparatide, it is reasonable to hypothesize a potential therapeutic role for abaloparatide in this clinical context.

The aim of this narrative review is to explore the potential role of abaloparatide in the management of osteoporosis in patients with CKD, particularly in stages G4–G5D. The review examines its mechanism of action, bone-targeted effects, and available data on efficacy and safety. Specific attention is given to the histomorphometric and microarchitectural changes, especially within the cortical compartment, alongside the clinical context of fracture risk and low bone turnover. Potential therapeutic indications and limitations of abaloparatide in this high-risk population are also discussed.

## 2. CKD Associated Osteoporosis and Fracture Risk

Osteoporosis is highly prevalent in patients with CKD, particularly in stages G4–G5D, with reported rates ranging from 20% to over 30%, depending on the study population and diagnostic criteria [5,6]. Early data from the general population revealed that up to 85% of elderly women (≥80 years) with osteoporosis had impaired renal function, highlighting the significant overlap between both conditions [18]. More recent studies have specifically addressed CKD cohorts. In a Chinese cross-sectional study including 502 patients with CKD stages G3–G5, the overall prevalence of osteoporosis by dual-energy X-ray absorptiometry (DXA) criteria was 31.7%, rising to 50% among patients with CKD stage 5 [5]. Imanishi et al. [6] similarly reported a prevalence of 32.3% in CKD stages G4–G5, considering lumbar spine and femoral neck BMD, identifying older age, female sex, lower body mass index (BMI), low vitamin D (calcidiol levels), and elevated PTH as key risk factors.

In the ERCOS study (the Spanish acronym for Enfermedad Renal Crónica y Osteoporosis), a multicenter Spanish cohort of 758 patients with CKD G4–G5, 38.3% of participants had osteoporosis, as defined by DXA or FRAX^®^ (Fracture Risk Assessment Tool), and 68% met the criteria for anti-osteoporotic treatment, although less than half received active therapy [19]. Hyun et al. [20] also found that low BMD was common in CKD, particularly in patients with sarcopenia, high sodium-potassium urinary ratio, and reduced physical activity. These studies consistently show that osteoporosis is underrecognized and undertreated in advanced CKD, despite its high prevalence and associated fracture risk.

The risk of fragility fractures, especially of the hip and vertebrae, is markedly elevated in this population [21,22,23], and additionally, compared to men, women showed 5–10 times more hip fractures [24,25]. CKD patients have a 1.4-to 9-fold increased risk of hip fracture compared to the general population, with the highest hazard ratios observed in patients over 80 years of age [6]. In a multicenter Spanish cohort (ERCOS study), 18.5% of CKD stage 4–5 patients had sustained at least one fragility fracture, with vertebral fractures being the most frequent (14.3%), followed by hip fractures (5.2%) and humeral fractures (3.7%) [19]. Other studies have confirmed a high incidence of femoral fractures in CKD. In a large U.S. cohort, the relative risk of hip fracture in CKD stages G3a–G5D was 2.36, and reached 7.66 in patients over 65 years [26]. Similarly, in a prospective cohort, patients with an estimated glomerular filtration rate (eGFR) < 60 mL/min/1.73 m^2^ had a 2-fold increased risk of hip fracture [23]. Beyond the increased incidence of fractures, their consequences in CKD are severe. Both femoral neck and vertebral fractures in patients with CKD are particularly associated with poor outcomes, including functional decline and postoperative complications [27]. In addition, hip fractures are associated with longer hospital stays and a high risk of institutionalization. In a recent meta-analysis, CKD was associated with a nearly 7-fold increase in 10-year postoperative mortality after hip fracture surgery [28]. These findings emphasize the need for systematic fracture risk assessment and early intervention in CKD stage G3, as well as stages 4−5, to prevent life-threatening skeletal events.

## 3. Renal Osteodystrophy (ROD)

As previously described, ROD represents the skeletal manifestation of CKD-MBD [1]. Traditionally, ROD has been classified into two main types according to bone remodeling activity: high-turnover and low-turnover disease. These categories reflect qualitative abnormalities of bone remodeling that may occur with quantitative changes in bone mass, which can be normal, increased (osteosclerosis), or decreased (osteopenia or osteoporosis) [1].

Within the spectrum of low-turnover bone disease, two subtypes are recognized: osteomalacia and ABD. The distinction between them is based on the presence or absence of mineralization defects (Table 2). Osteomalacia is characterized by defective mineralization, whereas ABD shows normal mineralization but markedly reduced bone formation. In addition, the KDIGO 2017 Clinical Practice Guidelines introduced a revised classification of bone abnormalities in CKD, based on three histomorphometric parameters: bone turnover, mineralization, and volume, known as the TMV System (Table 2). This classification provides a clinically relevant description of the underlying bone pathology, which helps define pathophysiology and guide therapy. Although PTH analogs have been primarily considered for adynamic bone disease, their potential role in other forms of low-turnover renal osteodystrophy may be relevant when standard therapy is insufficient to restore bone remodeling. It should be noted that it is currently considered that the inability to perform a bone biopsy should not preclude the use of antiresorptive therapy in patients at high risk of fracture.

ABD typically presents with relatively low PTH levels and diminished peritrabecular cellular activity. In this sense, ABD is a well-defined histological subtype of ROD, characterized by markedly suppressed bone turnover, diminished osteoblast activity, and minimal or absent bone formation, without mineralization defects. It is increasingly observed in patients with CKD stages G4−G5 and dialysis (G5D), especially among the elderly, diabetics, and those with hypercalcemia or excessively suppressed PTH levels [29,30,31]. Histologically, ABD is marked by low activation frequency, reduced Bone Formation Rate per Bone Surface (BFR/BS), and absent tetracycline labeling. This condition not only compromises bone strength and fracture healing but is also associated with vascular calcification and increased mortality.

**Table 2:** Histologic classification of renal osteodystrophy in CKD patients using the TMV system (adapted from Fusaro et al. [30]) and identification of potential PTH analog candidates based on the histologic subtype.

Type of Renal Osteodystrophy	Turnover	Mineralization	Volum	Potential PTH Analog Candidate *
Osteoporosis	Normal	Normal	Low	Yes
Adinamic bone disease	Low	Normal	Low to Normal	Yes
Osteomalacia	Low	Abnormal	Low to Medium	Not known
Osteitis fibrosa	High	Normal	Normal to High	No
Mixed osteodystrophy	Normal to High	Abnormal	Low to Normal	No

* Use of PTH analogs should be considered only after excluding hyperparathyroidism and other contraindications, and after obtaining informed consent. Their use in this context is off-label.

Retrospective analyses have currently identified ABD as the predominant subtype of ROD, with a reported prevalence of up to 60% in patients treated with hemodialysis [31]. The growing frequency of ABD has been attributed to the broader implementation of therapeutic strategies targeting secondary hyperparathyroidism and some related factors increasingly found in the CKD population, such as older age, malnutrition or systemic inflammation. Therefore, given the increasing prevalence of low bone turnover states, bone-forming agents may represent a particularly attractive therapeutic option, as they directly stimulate bone formation and could counteract the skeletal consequences of suppressed remodeling in these patients. Their use is further justified by the growing frequency of this condition in dialysis patients.

Bone biopsy remains the gold standard for assessing ROD in CKD, as it uniquely provides quantitative data on cortical thickness, trabecular and cortical bone volume, and turnover dynamics, information not reliably captured by biochemical markers [30,32]. However, several limitations (invasive, complex and time-consuming technique) prevent its implementation in routine clinical practice. Due to these obstacles, the updated KDIGO guidelines accepted that the inability to perform a bone biopsy may not justify withholding treatment from patients at high risk of fracture [10].

On the other hand, the recent controversies highlighted the systematic incorporation of bone turnover biomarkers (BTM) in guiding management of bone health [33], which seems to be helpful to distinguish the presence of high or low bone turnover in clinical practice. Briefly, BTM with non-renal clearance includes biointact PTH, bone-specific alkaline phosphatase (BALP), intact procollagen type I N-terminal propeptide (iPINP) and tartrate-resistant acid phosphatase isoform 5b (TRAP5b) [33,34]. The robustness of iPINP, BALP and TRAP5b in CKD patients undergoing dialysis has recently been shown, with minor fluctuations post-dialysis, whereas β-cross-linked C-terminal telopeptide of type I collagen (β-CTX) was markedly affected by dialysis [35].

The non-renal clearance of BTM shows high negative predictive values (≥90%) for discriminating between high- and low-turnover bone diseases in patients with advanced CKD [36], indicating their ability to identify both conditions [37]; in addition, these BTM also seem to be useful for predicting fracture risk, monitoring treatment response, and assessing the risk of treatment-related complications [34]. Thus, in advanced CKD, reduced cortical and trabecular bone volume, particularly in patients with low-turnover states, significantly contributes to skeletal fragility.

Thus, low values of non-renal clearance BTMs, including iPINP, BALP, and TRAP5b, together with low PTH values in individuals with CKD stages G4–G5D, indicate a low-turnover bone disease associated with CKD [37]. Indeed, the recently published consensus paper from multiple bone scientific societies recommends BALP and TRACP5b, measured by standardized assays, as reference BTMs for CKD-associated osteoporosis [34]. However, it should be noted that the availability of BTMs among nephrologists remains heterogeneous [38].

## 4. Bone Quality in Patients with CKD

In patients with CKD, particularly in stages G3 to G5 and those on dialysis, skeletal fragility emerges from more than just reduced BMD. The deterioration of bone quality, which is characterized by altered microarchitecture, cortical thinning and porosity, and impaired material strength, plays a central role in fracture risk, yet often remains undetected by conventional imaging. Although DXA is routinely used to assess BMD, it falls short in capturing these microstructural changes [8]. As this limitation became evident, researchers turned to advanced techniques to unravel the hidden aspects of bone deterioration in CKD. High-resolution quantitative computed tomography (HR-QCT), for instance, revealed striking cortical and trabecular impairments, particularly in patients with low-turnover bone disease [39,40]. Simultaneously, in vivo impact microindentation has revealed reduced bone material strength by detecting mechanical deficits that are not captured by densitometry [8]. Additionally, the trabecular bone score (TBS), derived from standard spine DXA, has emerged as a useful surrogate of trabecular integrity. In dialysis and transplant recipients, TBS values were consistently lower, offering additional insight where BMD remained inconclusive [39,41]. Still, none of these tools match the granularity of histomorphometry, the gold standard in assessing ROD. Through transiliac bone biopsy, clinicians can evaluate bone turnover, mineralization, and volume (TMV), and identify critical features such as cortical thinning and porosity, which are hallmarks of advanced CKD bone disease, particularly in adynamic bone states [30,32].

In this complex landscape, therapeutic decisions benefit from integrating structural information. Anabolic agents such as abaloparatide have shown the capacity not only to improve BMD, but also to enhance bone geometry and density distribution. Using 3D-DXA reconstruction, clinical trials have demonstrated that abaloparatide preferentially increases cortical volumetric BMD and improves femoral strength parameters compared to teriparatide [42,43]. These imaging results are consistent with histological findings showing preserved bone microarchitecture and the absence of mineralization defects in treated patients [44]. Altogether, these observations reinforce the need for a multidimensional approach to evaluate bone in CKD, one that looks beyond BMD alone and considers both structural and tissue-level determinants of fragility to guide individualized, mechanism-based therapies.

## 5. Abaloparatide: Mechanism of Action and Pharmacokinetic Profile

**Figure 1:**
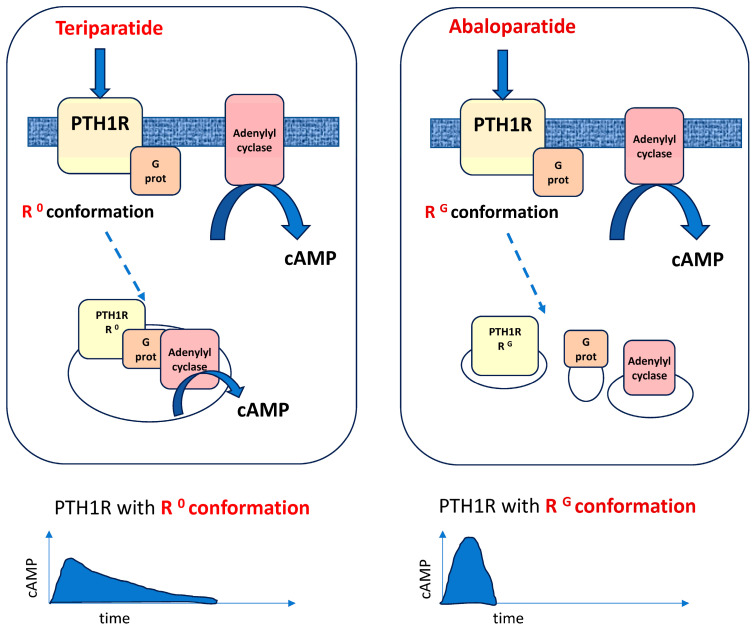
Teriparatide and abaloparatide bind to and activate the PTH1R but abaloparatide favors more anabolic configuration of the receptor. Teriparatide activates PTHR1 (a G-protein–coupled receptor expressed on osteoblasts and osteocytes) and stabilizes the R0 receptor conformation, resulting in the activation of adenylyl cyclase and a rapid increase in cAMP production. Subsequently, the hormone–receptor complex is internalized into early endosomes, where it continues to generate cAMP, leading to a sustained intracellular cAMP signal. Abaloparatide induces a similar signaling cascade and also mediates anabolic effects in osteoblasts; however, it preferentially stabilizes the RG receptor conformation. This conformation also activates adenylyl cyclase, producing a rapid rise in cAMP, but the hormone–receptor complex is internalized into distinct endosomal compartments with a shorter duration of cAMP delivery. Modified from Rachner et al. [46] and Tay et al. [47].

PTH, composed of 84 amino acids, regulates calcium metabolism through activation of the PTH receptor type 1 (PTH1R), a G protein–coupled receptor expressed on bone cells (osteoblasts and osteocytes) and in the kidney. Teriparatide and abaloparatide are PTH analogs that induce bone formation by activating PTH1R, which is expressed on bone cells [45]. Activation of PTH1R directly stimulates osteoblasts and osteocytes, enhances the differentiation of mesenchymal stem cells into osteoblasts, and prolongs osteoblast survival. In addition, PTH reduces sclerostin expression while simultaneously upregulating RANKL (Receptor Activator of Nuclear Factor κB Ligand), thereby coupling increased bone formation with enhanced osteoclast-mediated resorption. Teriparatide and abaloparatide are ligands that bind to and activate the PTH1R receptor but they appear to do so differently [45], as abaloparatide favors the transient, more anabolic configuration of the receptor (Figure 1).

Abaloparatide is a synthetic analog of PTH–related peptide (PTHrP [1,2,3,4,5,6,7,8,9,10,11,12,13,14,15,16,17,18,19,20,21,22,23,24,25,26,27,28,29,30,31,32,33,34]) that selectively binds and preferentially activates the RG conformation of PTH1R. It leads to a transient and rapid increase in cyclic AMP (cAMP) signaling. This results in enhanced osteoblast activity and bone formation, with relatively lower stimulation of bone resorption compared to teriparatide. The selective RG binding is associated with a favorable anabolic profile, characterized by stimulation of modeling- and remodeling-based bone formation and preservation of bone microarchitecture with lower cortical porosity and less hypercalcemia [46,47,48].

Pharmacokinetically, abaloparatide has a mean terminal half-life of approximately 1 h following subcutaneous injection. It is predominantly cleared via the renal route, primarily through glomerular filtration of peptide fragments, although active tubular secretion cannot be excluded [49]. According to the European Public Assessment Report (EPAR Eladynos) [50], systemic exposure to abaloparatide increases with declining renal function, with observed increases in maximum plasma concentration (C_max) of 3% to 44% in individuals with CKD. However, no pharmacokinetic studies have been conducted in patients with CKD stage G5D. These elimination characteristics are broadly comparable to those of teriparatide, which also exhibits a short half-life and renal clearance following subcutaneous administration [48].

## 6. Real-World Evidence on PTH Analogs in Osteoporotic Populations

Teriparatide and abaloparatide share anabolic properties but differ in their pharmacological profiles and regulatory approvals. Teriparatide is approved for the treatment of postmenopausal osteoporosis at high fracture risk, osteoporosis in men, and glucocorticoid-induced osteoporosis across most regions. Abaloparatide, in contrast, is approved in Europe exclusively for postmenopausal women at high risk of fracture, while in the United States and Japan, it is also approved for use in men with osteoporosis.

In the general population, clinical guidelines consistently recommend the use of PTH analogs in patients at very high or imminent risk of fracture. Most guidelines suggest initiating anabolic therapy under the following circumstances: recent fragility fractures (particularly if multiple, occurring during treatment with other antiosteoporotic agents, or involving severe vertebral or femoral fractures); low BMD, with thresholds varying among guidelines and typically defined as a T-score below −2.5, −3.0, or −3.5 standard deviations (SD); and elevated fracture risk based on FRAX^®^ scores, applying country-specific thresholds [51,52,53,54,55,56].

A recent panel of bone health experts recommended that, although definitions of very high fracture risk may differ across international guidelines, bone-forming agents should be considered first-line therapy in such cases. This position clearly supports the use of anabolic treatments as initial therapy in individuals with severe skeletal fragility [55]. However, CKD status is not addressed in these recommendations. Moreover, substantial differences in reimbursement criteria for bone-forming agents across countries may further contribute to the variability observed in clinical practice [55].

In parallel, the 2024 ASBMR (American Society for Bone and Mineral Research)/BHOF (Bone Health and Osteoporosis Foundation) Task Force Position Statement introduced updated recommendations for goal-directed treatment of osteoporosis [57]. The authors proposed treatment targets based on fracture risk and total femoral T-score, highlighting that increases in total femoral BMD are more strongly associated with fracture risk reduction than improvements at other skeletal sites [58]. Bone-forming agents were recommended as first-line therapy for patients with vertebral, femoral, and/or pelvic fractures—regardless of whether the fractures are recent or historical—or, in the absence of prior fragility fractures, for individuals with low BMD, defined as a lumbar spine T-score < −3.0 or a total hip T-score ≤ −2.8 SD. These thresholds were derived from baseline T-scores at which more than 50% of women achieved a T-score above −2.5 SD after approximately three years of anti-osteoporotic treatment [57].

All these publications and clinical guidelines emphasize the importance of treatment sequence. Initiating therapy with a bone-forming agent followed by an antiresorptive is more effective in preventing fractures than starting with an antiresorptive drug alone [55]. Thus, the sequence of osteoporosis treatment plays a critical role in optimizing patient outcomes, particularly in terms of improving BMD and reducing fracture risk.

## 7. Histomorphometric Analysis in Patients Treated with Abaloparatide

Histomorphometric analysis of bone biopsies has provided key insights into both the skeletal effects of abaloparatide and the diagnosis of ROD in patients with CKD. In patients treated with abaloparatide, early histological evaluation at 3 months demonstrated a consistent anabolic response, with significant increases in bone formation rate (BFR/BS) and mineralizing surface (MS/BS) across all bone envelopes (cancellous, endocortical, intracortical, and periosteal) as well as an elevated mineral apposition rate (MAR) specifically in the intracortical compartiment [59]. These findings reflect both remodeling- and modeling-based bone formation [59]. In the ACTIVE trial (Abaloparatide Comparator Trial In Vertebral Endpoints), long-term bone biopsies obtained at 12–18 months showed that static and dynamic histomorphometric indices revealed minimal differences between abaloparatide, teriparatide, and placebo. However, patients receiving abaloparatide exhibited reduced eroded surface, increased cortical porosity (also seen with teriparatide), and preservation of normal lamellar bone architecture without evidence of mineralization defects [44].

In this context, PTH analogs represent a biologically plausible and mechanistically targeted therapeutic option. Intermittent administration of agents such as teriparatide or abaloparatide may restore bone remodeling by stimulating osteoblast recruitment and reactivating quiescent remodeling surfaces. Unlike the catabolic effects of persistent endogenous PTH elevation in secondary hyperparathyroidism, exogenous analogs act through selective and transient activation of the PTH1R, promoting bone formation while minimizing resorption [32,60]. Furthermore, recent evidence suggests that static histomorphometry alone, even in the absence of tetracycline labeling, provides sufficient diagnostic accuracy to identify low-turnover states and inform individualized treatment strategies [61]. Therefore, in patients with histologically confirmed ABD and high fracture risk, PTH analogs may offer a promising approach to reverse low bone activity and improve skeletal outcomes.

## 8. PTH Analogs in CKD

Although bone-forming agents are widely recommended for patients at very high fracture risk in the general population, current osteoporosis guidelines provide limited guidance regarding their use in CKD, particularly in advanced stages. In fact, anabolic drugs are rarely used in the management of CKD-associated osteoporosis, as indicated by real-world data surveys [38]. Post hoc analyses from pivotal clinical trials have shown that PTH analogs (teriparatide and abaloparatide) exhibit similar efficacy in increasing BMD and reducing fracture risk in patients with normal renal function and those with CKD stages G1–G3 who have stable biochemical parameters, including non-elevated endogenous PTH levels [17,62].

**Figure 2:**
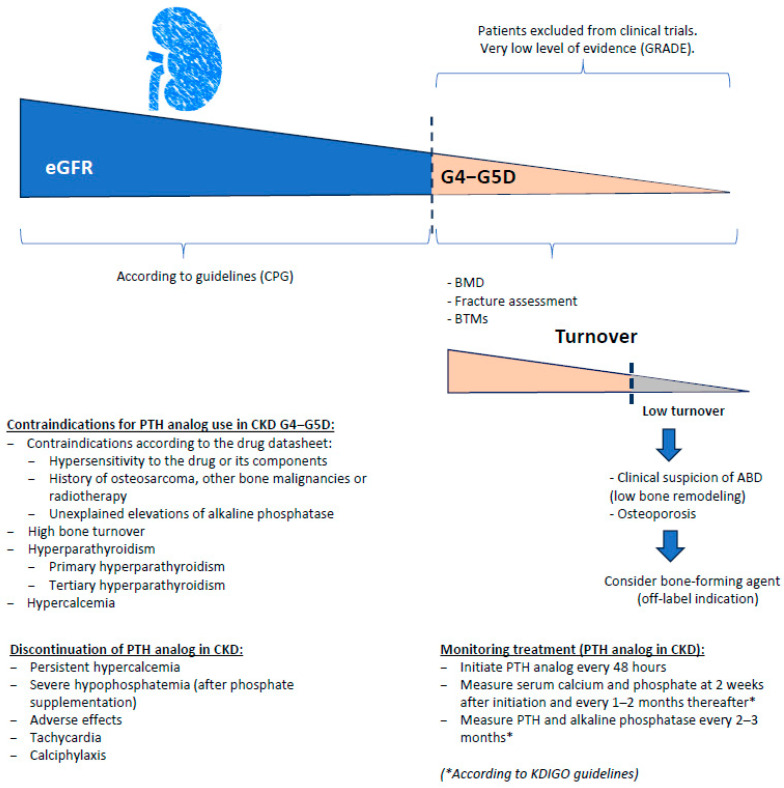
How and when to consider bone-forming agents in patients with stages G4–G5D. Figure created by the authors. Conceptual framework for considering bone-forming agents in patients with chronic kidney disease (CKD) stages G4–G5D. As kidney function declines, patients are increasingly excluded from pivotal clinical trials, resulting in a very low level of evidence (GRADE). In advanced CKD, bone assessment should include bone mineral density (BMD), fracture risk evaluation, and bone turnover markers (BTMs). In patients with low bone turnover and clinical suspicion of adynamic bone disease (ABD) and/or osteoporosis, the use of bone-forming agents may be considered as an off-label option. The figure summarizes contraindications, monitoring strategies, and discontinuation criteria for PTH analog therapy in this population. Data in accordance with KDIGO recommendations [4].

However, clinical data on the use of PTH analogs in CKD stages G4–G5D remain scarce. Most clinical trials have systematically excluded patients with severe renal impairment or dialysis dependence, leading to a significant evidence gap. Nonetheless, a few small studies suggest that teriparatide may be both effective and safe in patients undergoing hemodialysis therapy with histologically confirmed ABD, improving lumbar spine BMD without major adverse effects [63]. Similarly, real-world data indicate that abaloparatide may offer a favorable safety and fracture-risk reduction profile compared to teriparatide, without increased cardiovascular or renal complications [64,65]. It should be noted that part of the available real-world evidence derives from industry-sponsored sources, and this potential influence should be considered when interpreting these findings. Despite these promising findings, the use of PTH analogs in CKD stages G4–G5D should be limited to carefully selected cases, particularly those with high fracture risk and low bone turnover (Figure 2), and requires close monitoring of calcium, phosphate, and PTH levels, since isolated cases of calciphylaxis have been reported in subjects treated with PTH analogs [66,67].

From a clinical guidance perspective, the 2021 European Consensus Statement on the management of osteoporosis in CKD stages G4–G5D, jointly published by the European Renal Osteodystrophy (EUROD) workgroup and the International Osteoporosis Foundation (IOF), recommends fracture risk assessment based on clinical factors, BMD, FRAX^®^, and history of fragility fractures, with treatment decisions guided accordingly [14]. However, no specific recommendations on the preferred antiosteoporotic agents are provided. More recently, some experts have advocated for the use of bone-forming agents in patients with low bone turnover and high fracture risk, suggesting that this subgroup may derive particular benefit from anabolic therapy [68].

As detailed in Table 3, current clinical evidence on the use of teriparatide and abaloparatide in patients with advanced CKD (stages G4–G5 and G5D) remains limited. The table summarizes available studies, including trial design, CKD stage classification, treatment duration, effects on BMD, fracture incidence, and safety outcomes. Further research is needed to better define the efficacy, safety, and optimal therapeutic indications of PTH analogs in this high-risk population.

## 9. PTH Analogs and Hyperparathyroidism

CKD is frequently associated with secondary, tertiary, or even primary hyperparathyroidism. Persistently elevated serum PTH levels lead to substantial bone loss, particularly in the cortical compartment, thereby increasing fracture risk. In contrast, intermittent peaks of PTH activate the PTH1R, resulting in a significant increase in bone formation with comparatively less bone resorption—an effect mediated by synthetic PTH analogs such as abaloparatide [73]. This distinction is critical to understanding important limitations of PTH analogs in CKD-associated osteoporosis. Notably, PTH resistance (or hyporesponsiveness) has been described in patients with CKD stages G4–G5, particularly those on dialysis, many of whom exhibit low-to-normal bone turnover despite even markedly elevated PTH levels [14,74,75]. In this context, an animal model of CKD with hyperparathyroidism and hyperphosphatemia showed that daily teriparatide improved bone mineralization and volume, suggesting retained anabolic activity despite elevated endogenous PTH [14,76]. However, clinical evidence remains insufficient to support the routine use of PTH analogs in patients with overt hyperparathyroidism. According to current labeling, teriparatide is contraindicated in primary and tertiary hyperparathyroidism [77], while no specific contraindication is listed for abaloparatide in this context [50]. Both agents, however, are contraindicated in patients with severe CKD (eGFR < 30 mL/min/1.73 m^2^). Therefore, the off-label use of PTH analogs in patients with CKD should be restricted to carefully selected cases, based on individual fracture risk, bone turnover status, close clinical monitoring and when possible, with static and dynamic bone histomorphometric information.

## 10. PTH Analogs in CKD Stages G4–G5D: Safety Considerations

PTH analogs can be safely used, whenever properly indicated, in patients with CKD stage 3 and do not require dose adjustment in mild to moderate renal impairment (CKD G3) [62]. However, in patients with advanced CKD (stages G4–G5D), the use of PTH analogs such as teriparatide and abaloparatide remains off-label and requires individualized assessment due to limited data on long-term safety and efficacy.

Several small studies in patients on dialysis with histomorphometrically confirmed ABD have reported that teriparatide, administered daily or weekly according to the commercially available formulation in each country, can increase lumbar spine BMD without causing serious adverse events [60,70,71,72]. A Japanese post-marketing surveillance study also supported the tolerability of teriparatide in CKD stages G4–G5, though limited by small sample size and short duration [69]. Importantly, therapy with PTH analogs in this population requires exclusion of significant secondary or tertiary hyperparathyroidism, as elevated endogenous PTH levels may blunt the anabolic response. Careful monitoring of serum calcium, phosphate, and PTH is essential throughout treatment [12,14].

Evidence on abaloparatide in CKD stages G4–G5D remains scarce, as patients with severe renal impairment or dialysis dependence were excluded from pivotal trials. Nonetheless, post hoc analyses from the ACTIVE study revealed that abaloparatide was associated with lower rates of hypercalcemia compared to teriparatide, including in individuals with moderate renal impairment (CKD G3) [49,62,78]. In patients with baseline eGFR <60 mL/min, the absolute number of hypercalcemia events was 1/167 (0.6%) in the placebo group, 3/168 (1.8%) in the abaloparatide group, and 14/192 (7.3%) in the teriparatide group. When adjusted for treatment duration (18 months, equivalent to 1.5 years), these correspond to 0.40, 1.19, and 4.86 events per 100 patient-years, respectively. These data indicate that hypercalcemia was infrequent across all groups, although the incidence was higher with teriparatide compared with abaloparatide or placebo. Real-world data have further supported this favorable profile, showing no increase in renal or cardiovascular adverse events compared to teriparatide [64].

Regarding cardiovascular safety, abaloparatide demonstrated no excess risk of cardiovascular events, including myocardial infarction, stroke, or arrhythmia, in the ACTIVE trial [49]. Similar results were found in patients with renal impairment (CKD G3), in whom cardiovascular outcomes were comparable to placebo and teriparatide [62]. No increased cardiovascular risk has been detected in post-marketing or real-world studies [64]. Since patients with advanced CKD are inherently at high cardiovascular risk, PTH analogs, which have demonstrated a favorable cardiovascular safety profile, represent a reasonable alternative to romosozumab [79] as an anabolic therapy in individuals at very high risk of fracture.

However, PTH induces vasodilatory effects through activation of PTH1R on vascular smooth muscle and endothelial cells, leading to increased cAMP and nitric oxide production, which may cause transient hypotension. In patients undergoing hemodialysis, hypotension is a frequent event due to hemodynamic shifts and impaired vascular tone (e.g., diabetes, arteriosclerosis), requiring careful consideration, especially with a weekly administration schedule [60]. Although orthostatic hypotension associated with abaloparatide was reported in up to 17.1% of patients, compared with 15.5% with teriparatide and 16.4% in the placebo group, in the ACTIVE trial [78], in patients with baseline eGFR <60 mL/min, the absolute numbers corresponded to 0.00, 2.38, and 2.08 events per 100 patient-years for placebo, abaloparatide, and teriparatide, respectively. No specific analysis has been performed in the context of dialysis patients, and blood pressure should be carefully monitored. Moreover, although evidence on chronotherapy in this clinical setting is limited, medications in patients undergoing hemodialysis are recommended to be administered after the dialysis session. In addition, a higher incidence of palpitations was observed with abaloparatide (5.1%) compared with teriparatide (1.6%) and placebo (0.4%) in the ACTIVE trial, with events generally mild and rarely serious [49]. Serious palpitations were uncommon across all groups (≤0.1%) [49], and discontinuations due to palpitations occurred in 0.9% of abaloparatide-treated participants. Thus, patients with CKD G5D receiving abaloparatide should undergo close monitoring of heart rate after the first doses.

Nevertheless, the absence of randomized controlled trials in patients with CKD stage G5 or those on dialysis underscores the need for caution. In this high-risk population, PTH analogs should be reserved for selected cases, particularly those with biopsy-proven low bone turnover and high fracture risk, and always accompanied by close clinical and biochemical monitoring, as recommended in Figure 2 [4,29,32,63].

## 11. Conclusions

Patients with chronic kidney disease (CKD) face a markedly increased risk of osteoporosis and fragility fractures but remain underrepresented in clinical research and undertreated in practice. Although bone-forming agents effectively reduce fracture risk in the general population, evidence in CKD, particularly stages G4–G5D, remains scarce. Among available therapies, abaloparatide demonstrates a favorable anabolic profile, with lower calcemic effects than teriparatide, and potential suitability for patients with low bone turnover, including those with suspected adynamic bone disease (ABD). Emerging histomorphometric, imaging, and real-world data, some of which derive from industry-sponsored studies, suggest that abaloparatide may help preserve bone microarchitecture and improve skeletal outcomes in selected patients. However, its use in advanced CKD remains off-label and warrants careful monitoring. Further studies are needed to establish the safety, efficacy, and optimal use of PTH analogs in this high-risk population. A multi-disciplinary approach is also advised, including rheumatologist or bone specialist, nephrologist, radiologists, nutritionist and physical activity professionals. Until then, individualized therapy guided by bone turnover assessment and fracture risk evaluation is essential to address the persistent treatment gap in CKD-related osteoporosis.

## Figures and Tables

**Table 1:** Current staging of CKD (adapted from KDIGO [4]).

Stage	Description	eGFR (mL/min/1.73 m^2^)
G1	Kidney damage with normal or high GFR	≥90
G2	Kidney damage with mildly decreased GFR	60–89
G3a	Mildly to moderately decreased GFR	45–59
G3b	Moderately to severely decreased GFR	30–44
G4	Severely decreased GFR	15–29
G5	Kidney failure (End-stage renal disease if requiring dialysis/transplant)	<15

eGFR = estimated glomerular filtration rate by formula.

**Table 3:** Use of PTH Analogs in Advanced CKD (G4–G5) and Dialysis (G5D): Evidence from Clinical Trials and Real-World Data.

Author, Year	Drug	Type CKD	Number of Patients	Control Group	Dose of PTH Analog	Duration of PTH Analog	Lumbar BMD	Femur BMD	Fracture Incidence	Safety
Nishikawa et al., 2016 [69]	Teriparatide	CKD 4	30	–	20 μg/day	24 months	↑ BMD overall	↑ BMD (trend)	1 new fracture (CKD 5)	No serious AEs (4 mild in 33 patients)
		CKD 5	3	–	20 μg/day	24 months	↑ BMD (trend)	–	–	–
Cejka et al., 2010 [70]	Teriparatide	CKD 5	7	–	20 μg/day	6 months	↑ BMD lumbar (significant)	↑ BMD (not significant)	Not reported	No severe AEs reported
Mitsopoulos et al., 2012 [71]	Teriparatide	CKD 5	9	Yes	20 μg/day	13–16 months	↑ BMD lumbar (+4.9%)	↑ BMD femoral neck (+2.7%)	Not reported	No significant AEs
Sumida et al., 2016 [72]	Teriparatide	CKD 5	22	Yes (n = 8)	56.5 μg/week	48 weeks	↑ BMD lumbar (+3%)	No change	Not reported	10/22 discontinued due to AEs (transient hypotension)
Yamamoto et al., 2020 [60]	Teriparatide	CKD 5	10	Yes (n = 5)	56.5 μg/week	12 months	↑ BMD lumbar (+2.5% at 12 M)	No change	Not reported	40% dropout due to AEs (mainly hypotension)
Bilezikian et al., 2019 [62]	Abaloparatide	CKD 2	Included in trial	Yes	80 μg/day	18 months	↑ BMD, similar efficacy to CKD 1	↑ BMD	↓ fracture risk	Lower hypercalcemia vs. teriparatide in CKD 3 (3.6% vs. 10.9%)
		CKD 3	Included in trial	Yes	80 μg/day	18 months	↑ BMD, similar efficacy to CKD 1	↑ BMD	↓ fracture risk	No renal calcifications; well tolerated

AEs: Adverse events; BMD: Bone mineral density; ↑: Increase; ↓: Decrease. The level of evidence is very low (GRADE); thus, quantitative estimates should be interpreted with caution.

## Data Availability

The original contributions presented in this study are included in the article. Further inquiries can be directed to the corresponding author(s).
